# Dental Age-Group Classification from Panoramic Radiographs Using Convolutional Neural Networks

**DOI:** 10.3390/diagnostics16121816

**Published:** 2026-06-12

**Authors:** Essraa Gamal Mohamed, Ahmed R. El-Saeed, Hanin Ardah, Marco Malak Fayek, Mohammed Kayed

**Affiliations:** 1 Mathematics and Computer Science Department, Faculty of Science, Beni-Suef University, Beni Suef 62511, Egypt; essraa.gamal@yahoo.com; 2 Department of Mathematics and Statistics, College of Sciences, Imam Mohammad Ibn Saud Islamic University (IMSIU), Riyadh 11432, Saudi Arabia; 3 Department of Computer Sciences, College of Computer and Information Sciences, Princess Nourah bint Abdulrahman University, P.O. Box 84428, Riyadh 11671, Saudi Arabia; 4 Oral and Maxillofacial Radiology Department, Faculty of Dentistry, Minia University, Minia 61111, Egypt; 5 Computer Science Department, Faculty of Computers and Artificial Intelligence, Beni-Suef University, Beni Suef 62511, Egypt

**Keywords:** dental age, panoramic radiographs, convolutional neural network, forensic science

## Abstract

**Background/Objectives**: Determining chronological age is important in several domains, including forensic identification, clinical decision-making, legal matters, and immigration procedures. Dental tissues are widely recognized as reliable indicators of age because they undergo gradual and measurable structural changes throughout life. Nevertheless, most conventional dental methods show limited reliability when applied to adults and elderly individuals. The objective of this study was to investigate an automated deep learning-based approach for age-group classification in adults and seniors using panoramic dental radiographs. **Methods**: Panoramic dental radiographs were analyzed using a custom-designed Convolutional Neural Network (CNN) along with several established pre-trained deep learning architectures. The dataset consisted of 1469 radiographic images obtained from Egyptian individuals aged between 25 and 70 years. Images were classified into five predefined age categories using a classification-based framework, and the models were trained to learn age-related dental patterns from radiographic images. **Results**: The proposed Custom CNN achieved the highest accuracy of 85.2%, outperforming YOLOv8 (79.1%) and all other evaluated models, with the lowest prediction error (MAE = 1.92 years; RMSE = 5.46 years). Overall, the deep learning models demonstrated strong performance in classifying dental age groups, particularly within adult and senior populations, where conventional methods often show reduced reliability. **Conclusions**: The findings suggest that deep learning analysis of panoramic dental radiographs may serve as a supportive tool for age-group classification in adult populations, complementing rather than replacing traditional assessment methods. These results, while promising, are limited to the dataset and experimental conditions of this study, and broader applicability requires further validation across diverse populations and settings.

## 1. Introduction

Determining chronological age plays a critical role in various fields, including forensic science, criminal investigations, clinical diagnosis, civil matters, and immigration. Owing to the strong relationship between chronological age and dental development, dental age is widely regarded as a reliable indicator for age estimation. Tooth development and eruption progress through well-defined stages up to the completion of the third molar, reflecting a clear association between dental structures and age. However, once the third molar and its roots are fully developed, age estimation becomes considerably more challenging. In younger individuals, particularly those below the age of 25, age can be estimated with relatively higher accuracy based on distinct developmental stages of tooth eruption. In contrast, after the completion of dental development, estimation relies mainly on subtle morphological and degenerative changes that are less pronounced and more difficult to interpret. This complexity requires a clear understanding of the differences between primary and permanent dentition. Primary teeth, which number 20, are smaller, lighter, and characterized by larger pulp chambers, and they appear early in life before being replaced around the age of 12. Permanent teeth, numbering 32, are larger and more robust, and they erupt gradually from approximately age 6 up to around 25, with shorter and thicker roots that provide stronger structural support [1]. Accordingly, the focus of this work is on age-group classification in adults, where accurate age-group determination is inherently more difficult. Dental age estimation can be performed using morphological, biochemical, and radiological approaches, with radiological techniques such as orthopantomography, cone beam computed tomography, and magnetic resonance imaging being widely used in both clinical and forensic contexts [1,2,3]. These methods are applied across multiple disciplines, including forensic science, dentistry, and orthodontics [4,5].

While panoramic radiographs (OPGs) are widely used due to their accessibility and prevalence in the literature, they have inherent limitations such as magnification, distortion, and anatomical superimposition, which may affect adult age estimation. In contrast, CBCT provides a more accurate three-dimensional assessment of dental structures but is less commonly used due to cost and limited availability. Consequently, large-scale CBCT datasets suitable for deep learning applications are difficult to obtain. Therefore, OPG images were used in this study to reflect practical clinical conditions and data availability.

Considering the critical importance of dental age estimation, achieving a high degree of accuracy in results and analyses is essential, as inaccuracies can have significant repercussions. Existing methods predominantly rely on manual measurements and assessments, which can lead to errors, particularly when estimating the ages of adults and seniors. Deep learning is increasingly recognized as a supportive tool for clinical decision-making, assisting practitioners by providing objective and reproducible assessments rather than replacing human expertise. These technologies offer a promising solution for enhancing the accuracy of age estimation by enabling automated feature extraction from radiographic images.

Most existing approaches rely on manual assessment, which may introduce variability and reduce reliability, particularly in adult and senior populations. In this context, deep learning has emerged as a promising approach for medical image analysis, enabling automated feature extraction from radiographic data and improving consistency in predictions [6,7,8]. This study investigates the application of deep learning to dental-age-group classification in adult and senior populations, a task of direct relevance to forensic odontology and clinical practice. It is important to note that the present work adopts a classification-based framework, in which panoramic radiographic images are assigned to one of five predefined age categories, rather than a regression-based approach that predicts exact chronological age. This distinction reflects both the structure of the available dataset and the practical requirements of the target application, where categorical age-group determination is clinically and forensically meaningful. Unlike traditional approaches that rely on manually extracted features, deep learning models can capture complex, non-linear relationships within imaging data, leading to improved performance in age-group classification tasks [9,10,11].

In this work, we evaluate several state-of-the-art convolutional neural network (CNN) architectures for dental age-group classification using panoramic radiographic images. CNNs have demonstrated strong performance in various image analysis tasks due to their ability to learn high-level spatial features efficiently [12,13,14]. A custom CNN model was developed and compared with multiple pre-trained models, including YOLOv8, VGG-16, MobileNet, DenseNet-121, NASNet-Mobile, ResNet, EfficientNetV2M, EfficientNetV2, Xception, and InceptionV3 [12,13,14,15,16].

The subsequent sections of the paper are structured as follows: Section 2 provides an overview of related work on dental age assessment methods. Section 3 describes the materials and methods, including the dataset, preprocessing pipeline, deep learning architectures, training strategy, and evaluation metrics. The experimental results are presented in Section 4. Section 5 discusses the findings and outlines the study limitations. Lastly, Section 6 presents the conclusion.

## 2. Related Works

Recent studies have proposed numerous methodologies for accurate dental age estimation, especially utilizing deep learning techniques.

Houssein, E.H. et al. [17] analyzed 1429 panoramic radiographs using CNN models such as AlexNet and ResNet-101. The extracted features classified patients into eight age categories (0–70 years) via several classifiers, including decision trees, K-NN, linear discriminants, and SVM. Results favored AlexNet features, with K-NN showing the highest performance.

Kim et al. [18] applied a deep neural network (ResNet-152) to 2025 dental radiographs of first molars, dividing participants into seven age categories from childhood to over 60 years. Using majority voting and AUC metrics, their method achieved remarkable accuracy (0.94–0.98). The limitation was reliance solely on first molars.

Milošević, D. et al. [19] utilized 4035 individual tooth X-rays, covering ages 19–90. Models trained on entire panoramic images, targeted regions, and individual teeth tested various CNNs, including DenseNet201, Inception, ResNet variations, VGG models, and Xception. VGG16 achieved the best results. Similarly, Cular et al. [20] proposed combining ASM and AAM to identify dental contours on panoramic X-rays from 203 subjects. The extracted features, processed via neural networks, yielded a mean absolute error of 5.00 and 2.48 years.

Vila-Blanco, N. et al. [21] introduced automatic methods DANet and DASNet for chronological age estimation from OPG images of individuals aged 4.5 to 89.2 years. DANet employs convolutional-pooling layers, while DASNet integrates an additional gender-predictive path, significantly improving overall estimation accuracy, especially for younger individuals. Kahaki, S.M. et al. [22] also employed DCNNs on panoramic radiographs, focusing on molars of Malaysian children aged 1–17 years, achieving highly accurate age prediction.

Galibourg et al. [23] conducted a study comparing the Demirjian and Willems methods with ML regression models for age estimation in subjects aged between 2 and 24 years. The findings revealed that all machine learning models based on the developmental stages defined by Demirjian demonstrated higher accuracy in dental age estimation.

Guo, Y.C. et al. [24] contrasted conventional Demirjian-based manual estimation with CNN approaches for legal age determination (5–24 years). They employed EfficientNet and SE-ResNet 101, emphasizing robustness through optimized depth, width, and channel correlations. Results significantly favored CNNs over manual methods, revealing differences in human and machine feature extraction. Zhang, Q. et al. [25] developed a deep learning-based method (ResBlocks) for age estimation using pulp chamber segmentation from 3D CBCT scans of first molars, covering ages 10–60. Their approach enabled rapid, accurate volume-based age estimations. Farhadian, M. et al. [26] examined neural networks against regression models to estimate age using canine pulp-to-tooth ratios from 300 CBCT images (14–60 years). Results demonstrated that neural networks outperformed regression, with substantially lower RMSE (4.40 vs. 10.26) and MAE (4.12 vs. 8.17). The neural network had its lowest accuracy among the oldest participants, whereas the regression model performed poorly among younger participants.

Previous studies have employed both two-dimensional (OPG) and three-dimensional (CBCT) imaging modalities for dental age estimation. CBCT-based approaches enable volumetric analysis, such as pulp-to-tooth ratio and pulp chamber volume, which are considered more accurate for adult age estimation. However, OPG-based methods remain more widely used in clinical practice and research due to their accessibility and availability [1]. It is also important to distinguish between age estimation in children and adults. In pediatric populations, age estimation is primarily based on developmental stages of teeth, whereas in adults, it depends on degenerative and morphological changes. These represent fundamentally different biological processes and should be considered when interpreting results. Furthermore, methodological differences between imaging modalities and evaluation approaches should be acknowledged. Two-dimensional methods rely on projected anatomical features, whereas three-dimensional techniques provide volumetric information. In addition, classification-based approaches, as adopted in this study, differ from regression-based methods, as they predict age groups rather than exact chronological age, reflecting different evaluation frameworks.

## 3. Materials and Methods

This section describes the approach followed in this study, including details of the dataset, image acquisition and preprocessing, model construction, and experimental procedures.

### 3.1. Dataset

In this study, the analysis was limited to permanent teeth, as age-group classification becomes considerably more challenging following the completion of dental development, at which point estimation relies primarily on subtle morphological and degenerative changes rather than distinct developmental stages. The dataset consists of 1469 panoramic dental X-ray images collected from male and female patients aged between 25 and 70 years. These radiographic images were obtained from the outpatient clinic at the Faculty of Dentistry, Minia University, Egypt, reflecting authentic real-world clinical conditions. Given the retrospective nature of the study and its exclusive reliance on pre-existing archived radiographic images, no new clinical interventions or patient interactions were involved. The images were grouped into five predefined age categories, as presented in Table 1. The study protocol was reviewed and approved by the Research Ethics Committee of the Faculty of Dentistry, Minia University, Egypt (Approval No. 533/2021; Committee Meeting No. 83; dated 1 November 2021). The study was conducted in full accordance with the ethical principles outlined in the Declaration of Helsinki. The data were handled in a fully anonymized manner, ensuring that no personal or sensitive information could be traced back to individual patients. Radiographs were included if they exhibited acceptable image quality and adequate visibility of dental structures within the defined age range. Images were excluded if they exhibited severe artifacts, significant anatomical superimposition, or pathological conditions that would substantially compromise the visibility of dental structures relevant to age-group classification. Specifically, cases presenting with extensive multi-surface restorations obscuring root morphology, complete endodontic treatment of multiple teeth with post-and-core restorations, advanced generalized periodontal bone loss exceeding 50% of root length, or multiple missing posterior teeth resulting in insufficient dental representation were excluded from the dataset. In contrast, images presenting with limited single-surface restorations, minor periapical changes, or mild-to-moderate localized bone loss were retained, provided that the panoramic image quality remained diagnostically acceptable and the dental structures relevant to age-group classification—including tooth crown morphology, root configuration, pulp chamber dimensions, and alveolar bone patterns—remained clearly visible. This approach was intentional, as retaining such clinically realistic variability enhances the generalizability of the trained models to real-world conditions, while ensuring that the primary features used for age-group determination remained interpretable across all retained images.

### 3.2. Data Preparation and Processing

Panoramic radiographs were acquired using a SCANORA^®^ 3Dx dental unit (Soredex, Helsinki, Finland) under standardized clinical conditions, with a scan time ranging from 18 to 34 s, an effective exposure time between 2.4 and 6 s, a focal spot size of 0.5 mm, and exposure parameters within the range of 60–90 kV and 4–10 mA. To ensure consistency across all samples, patient positioning was carefully controlled during image acquisition. A midline laser was used to align the center of the face, while a Frankfort horizontal laser line was adjusted parallel to the floor. Patients were instructed to maintain an upright posture and a forward gaze during imaging. All radiographs were obtained following routine clinical procedures and were reviewed to ensure acceptable image quality and diagnostic usability. Prior to model input, the region of interest (ROI) was defined as the full panoramic dental image, including both the upper and lower jaws, without applying manual cropping. This approach allows the models to learn from the complete anatomical context of dental structures.

Effective data preprocessing plays a critical role in preparing input data for reliable model training. This stage involves standardizing the data through normalization and scaling, and reducing potential noise and inconsistencies in the images. Such preparation improves data quality and enables models to learn more robust and meaningful representations, ultimately enhancing prediction accuracy [27].

**Figure 1:**
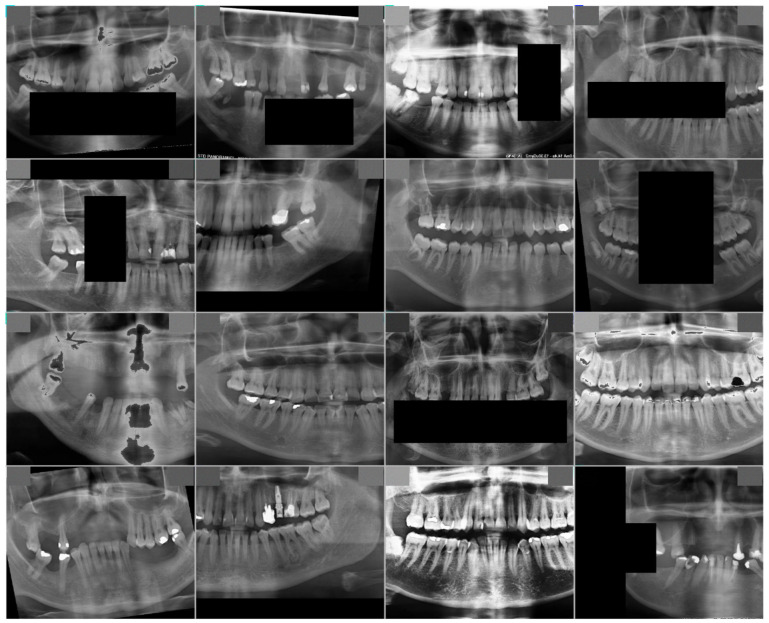
Examples of augmented panoramic radiographs used in training batches, illustrating the applied augmentation techniques including horizontal flipping, geometric transformations, brightness adjustment, channel shifting, and cutout.

To further strengthen the generalization capability of the models, a range of data augmentation techniques was applied during training. These included horizontal flipping, geometric transformations such as width and height shifting and zooming, as well as brightness adjustment and channel shifting [28,29]. In addition, the cutout technique was applied to improve model robustness by encouraging the network to focus on distributed features across the image. All augmentation operations were applied uniformly across all models to ensure a fair comparison. A detailed summary of these techniques is provided in Table 2. An illustrative example of the applied data augmentation techniques on panoramic radiographs is shown in Figure 1. Furthermore, mixed-precision training was employed to enhance computational efficiency and reduce memory consumption during the training process. Input image dimensions were selected based on the architectural requirements of each model. For the pre-trained models—including YOLOv8, VGG-16, DenseNet-121, MobileNet, Xception, InceptionResNetV2, NASNetMobile, InceptionV3, ResNet, and EfficientNetV2M—images were resized to 640 × 640 pixels, preserving a high level of spatial detail and ensuring compatibility with the models’ original input specifications. For the Custom CNN model, which was designed as a lightweight architecture trained from scratch on the available dataset, an input resolution of 64 × 64 pixels was adopted following empirical experimentation. This choice was motivated by the need to balance feature extraction capability with the risk of overfitting inherent to training a relatively simple architecture on a dataset of 1469 images. Despite this spatial downsampling, the model demonstrated strong classification performance, suggesting that age-discriminative features in panoramic radiographs—such as overall tooth morphology, relative root-to-crown proportions, and generalized alveolar bone patterns—remain distinguishable at this resolution after appropriate preprocessing and augmentation.

### 3.3. Deep Learning Models

Convolutional neural networks (CNNs) are a class of feed-forward neural networks that have achieved remarkable success in image recognition and classification tasks due to their ability to automatically learn hierarchical feature representations from raw image data [30]. These networks operate by extracting relevant features through successive transformations, where redundant information is reduced into a more compact and meaningful representation of the input data [31]. A typical CNN architecture consists of three main components: convolutional layers, pooling layers, and fully connected layers. Convolutional layers perform weighted operations followed by nonlinear activation functions such as the rectified linear unit (ReLU), defined in Equation (1) , enabling the network to capture complex patterns within the data. Pooling layers reduce the spatial dimensionality of feature maps, helping control overfitting and improve computational efficiency. Finally, fully connected layers aggregate the extracted features to perform classification in the output layer.

(1) ReLu(x)=max(0,x)

The effectiveness of CNNs lies in their ability to capture spatial relationships and discriminative patterns within images, making them particularly well-suited for medical image analysis [32]. In the context of dental radiographic images, CNNs can efficiently learn subtle structural variations in dental anatomy that are associated with age-related changes. This capability makes them an appropriate choice for dental age-group classification, where accurate feature extraction from complex radiographic patterns is essential.

#### 3.3.1. Pre-Trained Deep Learning Models

Building on the strengths of convolutional neural network (CNN) architectures, pre-trained deep learning models have been widely adopted to enhance performance, particularly in scenarios with limited datasets. These models are initially trained on large-scale datasets such as ImageNet and can be effectively adapted to domain-specific tasks through transfer learning. This approach enables the models to leverage previously learned feature representations while being fine-tuned for dental radiographic analysis [24,25,26]. In this study, a set of well-established pre-trained models was selected to provide a comprehensive evaluation of different architectural strategies. These models were chosen based on their proven performance in image classification tasks and their ability to capture diverse feature representations relevant to dental age estimation. The evaluated models are described as follows:

YOLOv8 is a modern deep learning architecture that supports both object detection and classification tasks. It is characterized by its high speed and strong prediction accuracy, making it suitable for real-time applications [33]. In this study, it was adapted for classification by modifying the output layer to predict five age groups. It was selected for its ability to capture both global and local image features efficiently.

VGG-16 is a deep convolutional neural network consisting of 16 layers, known for its simple and uniform architecture. It utilizes small convolutional filters that enable effective hierarchical feature extraction [34]. It was included as a baseline model due to its stability and well-established performance in image classification tasks.

MobileNet is designed for lightweight and efficient computation, especially in mobile and embedded environments. It uses depthwise separable convolutions to significantly reduce model complexity while maintaining accuracy [35]. It was selected to evaluate the performance of efficient models under limited computational resources.

NASNet-Mobile is an architecture generated using neural architecture search techniques, aiming to optimize both accuracy and efficiency [36]. It was chosen to represent automatically optimized network designs suitable for real-time applications.

ResNet introduces residual connections that help overcome the vanishing gradient problem, allowing the training of deeper networks [37]. It was selected for its strong capability in learning complex and deep feature representations.

Inception-ResNet v2 combines the strengths of Inception modules and residual connections, enabling multi-scale feature extraction with improved training stability [38]. It was included for its effectiveness in capturing complex patterns in medical images.

EfficientNetV2 is designed to optimize both model accuracy and computational efficiency through compound scaling of depth, width, and resolution [39]. It was selected for its ability to achieve high performance at reduced computational cost.

InceptionV3 employs inception modules that process multiple convolutional filter sizes in parallel, allowing the model to capture features at different spatial scales [40]. It was included to evaluate multi-scale feature extraction in dental radiographs.

Xception extends the Inception architecture by replacing standard convolutions with depthwise separable convolutions, improving efficiency and feature extraction capability [41]. It was selected for its ability to model complex image structures with fewer parameters.

DenseNet-121 is characterized by dense connectivity, where each layer is connected to all preceding layers, enhancing feature reuse and gradient flow [42]. It was included due to its efficiency in learning rich feature representations with fewer parameters.

All pre-trained models were initialized using ImageNet weights to leverage transfer learning. The original architectures were preserved, with modifications limited to the final classification layer to predict the five predefined age groups, as shown in Table 1. The selection of these models was motivated by the need to evaluate a diverse range of architectural strategies, including lightweight networks, deep residual networks, densely connected architectures, and multi-scale feature extraction approaches. This diversity ensures a comprehensive and fair comparison of model performance in dental-age group classification using panoramic radiographs.

#### 3.3.2. Custom CNN Model

A custom CNN model was developed to specifically address the characteristics of panoramic dental radiographs and the requirements of age-group classification. The model was designed with a relatively simple architecture to balance feature extraction capability and the limited size of the available dataset.

The architecture consists of three consecutive convolutional blocks. Each block includes a Conv2D layer with 32 filters and a kernel size of 3, followed by a ReLU activation function and a max-pooling operation. These layers enable progressive extraction of low-level to high-level features from the input images.

**Figure 2:**
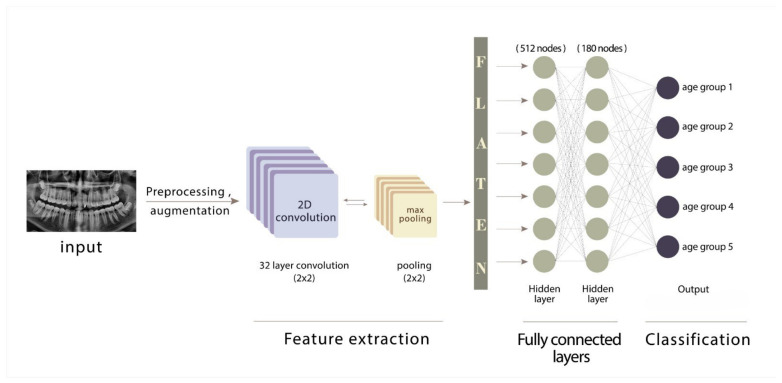
CNN architecture for the custom CNN model.

Following the convolutional layers, the feature maps are flattened and passed through fully connected layers composed of 512 and 180 neurons, respectively, both using ReLU activation. The final classification layer consists of five neurons with a softmax activation function, corresponding to the predefined age groups. The complete architecture of the proposed model is illustrated in Figure 2. The design of the Custom CNN model was guided by empirical experimentation and domain-specific considerations to ensure effective learning while avoiding excessive model complexity.

### 3.4. Training Strategy

To ensure a consistent and reproducible training process, all experiments were conducted using Python 3.10, PyTorch 2.3.1, and TensorFlow 2.16 with Keras 3.0 within a Google Colab environment with GPU support. Grad-CAM visualizations were generated using the tf-keras-vis library (version 0.8.5). The dataset was divided into training and validation sets, consisting of 1172 and 297 images, respectively. The distribution of the five age groups was preserved across both sets to ensure balanced representation and to minimize potential bias during training and evaluation. All pre-trained models were initialized using weights from the ImageNet dataset to leverage transfer learning. The original architectures were preserved, with modifications applied only to the final classification layer to match the five predefined age groups, as presented in Table 1. In contrast, the Custom CNN model was trained from scratch using the same dataset. To improve convergence, the learning rate was dynamically adjusted during training using the ReduceLROnPlateau strategy.

To reduce overfitting and improve generalization performance, several regularization techniques were applied. Early stopping was used to monitor validation loss and terminate training when no further improvement was observed, while restoring the best model weights. In addition, model checkpointing was employed to save the optimal model during training. All models were trained under the same experimental conditions, including identical preprocessing and training configurations, to ensure a fair and unbiased comparison. The hyperparameter search space is presented in Table 3.

### 3.5. Evaluation Metrics

To provide a comprehensive assessment of model performance, multiple complementary evaluation metrics were employed [43]. Classification performance was evaluated using accuracy, precision, recall (sensitivity), and F1-score, reported both per age group and as macro- and weighted-averaged values across all five classes. These metrics jointly capture the model’s ability to correctly identify each age category while accounting for class imbalance in the dataset. In addition, to quantify the magnitude of misclassification errors in age units rather than mere class membership, two ordinal regression-style metrics were derived from the confusion matrices: the Mean Absolute Error (MAE) and the Root Mean Square Error (RMSE) [44], both expressed in years. For these calculations, each age group was represented by its midpoint (27.5, 35, 45, 55, and 65 years for the five categories, respectively), allowing the conversion of classification errors into a meaningful temporal scale. MAE and RMSE are computed as follows:

(2) $$\begin{array}{cc} \mathrm{MAE} & =\frac{1}{N}\left(\left|{\hat{y}}_{1}-y_{1}\right|+\cdots +\left|{\hat{y}}_{N}-y_{N}\right|\right),\\ \mathrm{RMSE} & =\sqrt{\frac{1}{N}\left[{\left({\hat{y}}_{1}-y_{1}\right)}^{2}+\cdots +{\left({\hat{y}}_{N}-y_{N}\right)}^{2}\right]}. \end{array}$$

where $y_{i}$ denotes the midpoint of the true age group; ${\hat{y}}_{i}$ denotes the midpoint of the predicted age group; and *N* is the total number of test samples. This combined evaluation framework provides both a categorical perspective (accuracy, precision, recall, F1-score) and a temporal-error perspective (MAE, RMSE), enabling a more rigorous and clinically meaningful assessment than accuracy alone.

## 4. Results

This section reports the performance of the proposed and evaluated deep learning models for dental age-group classification using panoramic radiographs. In line with the multi-metric evaluation framework described in Section 3.5, results are reported using accuracy, precision, recall, and F1-score, calculated both per age group and as overall macro- and weighted averages. A comparative analysis of the different model architectures follows, with detailed per-class behavior examined to identify strengths and weaknesses across the five age categories.

We employed the YOLOv8 architecture, recognized for its advanced image classification capabilities and optimized performance. As the latest iteration of the You Only Look Once model, YOLOv8 enhances classification through its modified backbone, CSPDarknet53, which improves feature extraction using Cross Stage Partial connections. The model incorporates a Path Aggregation Network (PANet) as its neck, facilitating effective multi-scale feature fusion. This architecture allows YOLOv8 to achieve an impressive balance between speed and accuracy, making it suitable for real-time applications such as age classification from dental X-rays.

For our experiments, we utilized the pre-trained yolov8x-cls.pt model, specifically designed for classification tasks. The implementation was carried out using the Ultralytics YOLOv8 framework (version 8.2.73). This model includes several convolutional layers, followed by fully connected layers and a softmax layer for multi-class classification. By initializing with pre-trained weights, it leveraged features learned from extensive image datasets. The model was trained on a dataset comprising 1172 dental images over 30 epochs, with a batch size of 16 and an input image size of 640 × 640 pixels. Optimization was performed using the AdamW optimizer, with a learning rate of 0.000714 and a momentum of 0.9. To enhance generalization, data augmentation techniques, including horizontal flipping, scaling, and color space transformations, were applied. Additionally, training was conducted with mixed precision to improve memory efficiency and computational speed, ensuring an optimal trade-off between accuracy and computational efficiency in classifying dental X-rays.

The comparative evaluation of image classification models for dental age-group classification revealed varying performance levels across the five predefined age categories. YOLOv8 achieved the highest validation accuracy of 79.1%, showcasing its robust performance attributed to its advanced architecture, including the CSPDarknet53 backbone and Path Aggregation Network (PANet). Following YOLOv8, VGG-16, despite being an older model, achieved a validation accuracy of 70%. Its deep network structure, consisting of 16 layers, continues to be effective for image classification tasks, demonstrating its enduring capability.

DenseNet-121 was applied next, achieving an accuracy of 67%. Its densely connected architecture allowed for efficient feature extraction and reuse throughout the network. MobileNet was also employed, achieving an accuracy of 65%, recognized for its lightweight design and computational efficiency. Xception, known for its depthwise separable convolutions, similarly reached an accuracy of 65%.

Inception-ResNet v2 achieved 60% accuracy in dental image classification. NASNet-Mobile was tested, achieving a lower accuracy of 59%. Although it features an adaptive architecture that optimizes model design, its performance was not as strong as that of MobileNet and Xception. InceptionV3 yielded an accuracy of 58%, showcasing a sophisticated architecture that captures multi-scale features, yet its performance was relatively modest.

ResNet achieved an accuracy of 57%, leveraging its deep residual learning framework to handle complex image data effectively, though its performance remained moderate. In contrast, EfficientNetV2 recorded the lowest accuracy at 49%. Despite its design for enhanced efficiency and performance, it struggled in this specific application.

Custom CNN model achieved the highest accuracy in our experiments for dental image classification, reaching 85%. To evaluate whether the predictions generated by the Custom CNN are grounded in anatomically and radiologically meaningful features rather than background noise or imaging artifacts, Grad-CAM [45] was applied to the final convolutional layer of the trained model across a representative sample of correctly classified images drawn from all five age groups. The resulting activation maps were systematically examined and correlated with established radiological markers of dental aging recognized in the forensic odontology and dental radiology literature. Three anatomical regions of consistent and prominent activation were identified across the analyzed sample. The first and most dominant activation site corresponds to the pulp chamber and coronal root canal region. Radiologically, progressive narrowing of the pulp chamber resulting from the continuous deposition of secondary and tertiary dentin throughout adult life is one of the most extensively documented and quantified indicators of dental age, forming the anatomical basis of multiple validated forensic aging methods. The consistent localization of model activation in this region, therefore, provides direct biological justification for the learned feature representations and supports the radiological validity of the classification decisions. The second region of notable activation encompasses the mid-root dentinal wall, where increasing radiodensity associated with progressive peritubular mineralization and secondary dentin apposition represents a recognized histological and radiographic correlate of advancing age. Activation in this region was more prominent in images from the older age categories, consistent with the greater degree of dentinal sclerosis expected at advanced ages. The third activation region, observed predominantly in images classified into the 50–60 and 60–70 age groups, corresponds to the alveolar bone crest and surrounding trabecular architecture. Age-related reduction in alveolar bone height and changes in trabecular density and pattern are established supplementary radiological markers used in forensic age assessment, and their prominence in the model’s activation maps for older age categories is radiologically coherent. Minimal activation was observed in radiologically non-informative regions, including the surrounding soft tissues, mandibular condyles, and image background areas. This spatial selectivity supports the conclusion that the model has acquired a feature representation consistent with clinically and forensically relevant dental anatomy, thereby providing a meaningful basis for the interpretability claims made in this study.

**Figure 3:**
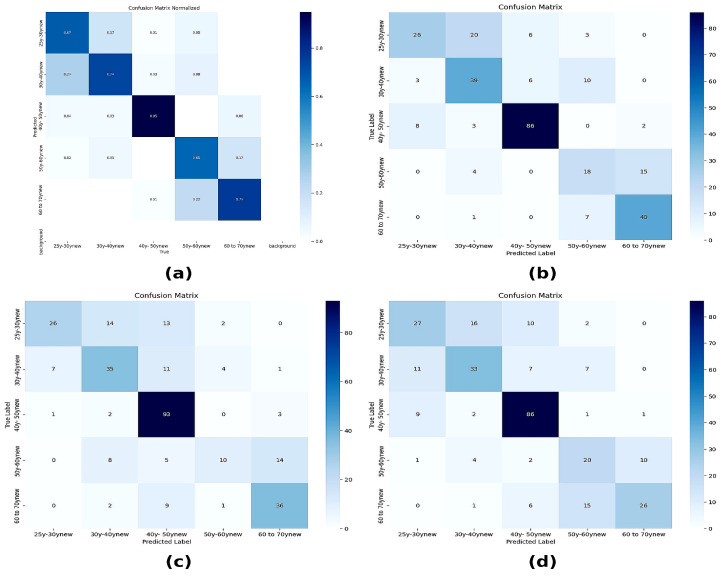
The confusion matrix for some of the proposed models: (**a**) YOLOv8, (**b**) VGG, (**c**) DenseNet121, and (**d**) MobileNet.

Deep learning models, including Convolutional Neural Networks, are commonly assessed using performance metrics derived from a confusion matrix. This matrix offers a detailed comparison of the model’s predictions against the actual class labels. Key performance metrics calculated from the confusion matrix include recall, or sensitivity, which quantifies the model’s ability to accurately identify positive instances among all actual positives. Precision, also known as positive predictive value, measures the model’s accuracy in predicting positive instances from all predicted positives. The F1-score integrates both recall and precision, providing a comprehensive evaluation of the model’s performance by accounting for both false positives and false negatives. This metric is particularly valuable in situations where both types of errors are critical. Accuracy, a fundamental metric, assesses the overall correctness of the model’s predictions by determining the ratio of correctly classified instances to the total number of instances. These metrics, derived from the confusion matrix, offer essential insights into the performance of deep learning models, helping researchers and practitioners evaluate their effectiveness and appropriateness for various tasks. The confusion matrix for selected models with high accuracy is presented in Figure 3. Additionally, a comparison of the dataset results for all models in our experiments is provided in Table 4.

**Figure 4:**
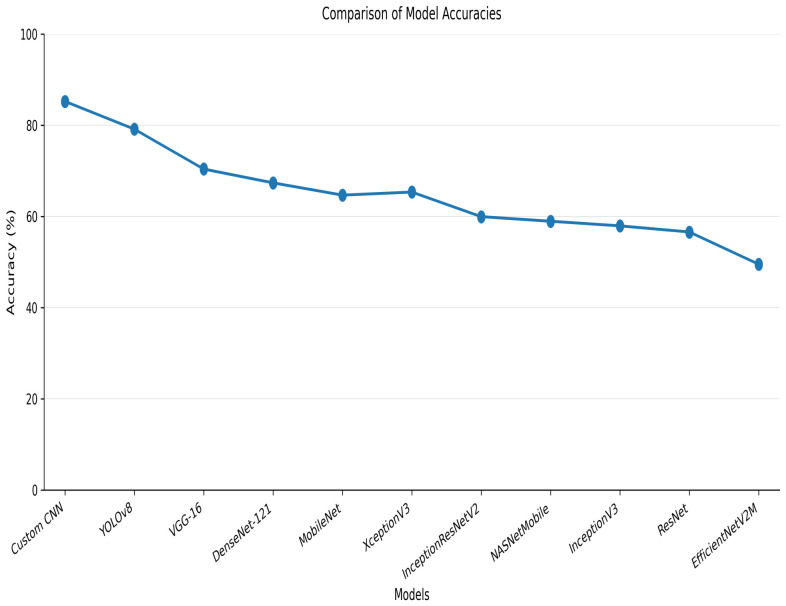
Comparison of accuracy percentages for all models in our experiment.

The performance metrics (Precision, Recall, F1-score, and Support) for various models are compared in detail, highlighting the differences across different age groups. Models such as YOLOv8, VGG-16, DenseNet-121, MobileNet, Xception, InceptionResNetV2, NASNetMobile, and InceptionV3 were evaluated. Their performance is analyzed over five age groups: 25–30, 30–40, 40–50, 50–60, and 60–70. Precision, Recall (Sensitivity), F1-score, and the number of samples (Support) are presented for each age group, providing insights into the models’ classification effectiveness. Yolo8 and VGG-16, for example, tend to show high accuracy in the 40–50 age group, while other models exhibit varying performance across different age ranges, as shown in Table 5. Finally, the Custom CNN model’s performance on the testing dataset is summarized in Table 6, showcasing its strong results in age classification. The model achieved Precision scores ranging from 0.82 to 0.87 and Recall rates between 0.80 and 0.86 across different age groups. With an overall accuracy of 0.85, the Custom CNN model demonstrates high accuracy compared to other models, making it effective for classifying age ranges. Figure 4 illustrates the accuracy percentages of various models evaluated on a testing dataset. Among the models compared, the Custom CNN achieved the highest accuracy, reaching approximately 85.20%. YOLOv8 follows with an accuracy of 79.10%. To further strengthen the evaluation, the Mean Absolute Error (MAE) and Root Mean Square Error (RMSE) were computed for all models using the age-group midpoints as ordinal targets, expressing prediction errors in years rather than class units. As reported in Table 7, the Custom CNN achieved the lowest error magnitudes (MAE = 1.92 years; RMSE = 5.46 years), followed by YOLOv8 (MAE = 2.47 years; RMSE = 6.00 years). The remaining pre-trained models showed progressively larger errors, ranging from MAE = 3.91 years for VGG-16 up to MAE = 8.44 years for EfficientNetV2M. These results confirm that the Custom CNN not only achieves the highest classification accuracy but also produces predictions with the smallest temporal deviation from the true age group, indicating that the majority of its misclassifications occur in immediately adjacent age categories rather than distant ones. This pattern is clinically and forensically desirable, as it limits the magnitude of error in age-group estimates.

A detailed examination of per-age-group classification performance, as reported in Table 5 and Table 6, reveals a consistent pattern across all evaluated architectures that warrants explicit discussion. Classification performance is highest for the 40–50 age group in virtually all models, with F1-scores of 0.87, 0.94, and 0.87 for the Custom CNN, YOLOv8, and VGG-16, respectively. This age category is the most numerically represented in the dataset and corresponds to a range in which multiple radiologically distinguishable features—including moderate but clearly visible pulp chamber reduction, established root canal dimensions, and early alveolar bone changes—provide sufficient inter-group contrast to support reliable automated classification. In contrast, classification performance declines progressively for the 50–60 and 60–70 age categories across the majority of evaluated models. For the 50–60 group, the Custom CNN achieves an F1-score of 0.81, while architectures such as DenseNet-121 and MobileNet record F1-scores of 0.37 and 0.49, respectively, representing a substantial reduction relative to their performance on the 40–50 group. This decline reflects the combined effect of a smaller number of training samples available for this class and the inherently reduced radiological distinctiveness of age-related dental changes in this interval. In the 60–70 age category, performance partially recovers in several models, likely attributable to the more pronounced macroscopic changes at this extreme of the age range, including generalized advanced tooth wear, extensive secondary dentin deposition, visible as marked pulp chamber obliteration, and more clearly defined alveolar bone remodeling patterns. These observations highlight the fundamental challenge of automated dental age classification in older adult populations, where the biological rate of change is reduced, inter-individual variability is high, and the boundaries between adjacent age categories become increasingly indistinct from a radiographic standpoint.

## 5. Discussion and Limitations

The literature on dental age estimation demonstrates considerable variability in methods and approaches, reflecting the absence of standardized protocols and consensus within the field [17,18,19]. Different studies rely on various teeth, such as molars and canines, and employ multiple imaging modalities, including CBCT, orthopantomography (OPG), and MRI, which contributes to heterogeneity in reported outcomes [22,23,24]. In addition, many researchers select specific tooth subsets based on the targeted age group, aiming to identify correlations that enhance estimation accuracy across different populations [25,26]. This variability highlights the complexity of dental age estimation and the need for more robust and standardized methodologies, particularly with the growing adoption of deep learning approaches. An important methodological consideration is the distinction between classification-based and regression-based approaches in dental age assessment. Regression-based methods predict a continuous chronological age value and are typically evaluated using Mean Absolute Error (MAE) and Root Mean Square Error (RMSE). In contrast, the classification-based framework adopted in this study predicts discrete age-group membership and is primarily evaluated using accuracy, precision, recall, and F1-score. The choice of a classification framework is justified by the forensic and clinical context targeted in this study, where age-group determination is the operationally meaningful outcome. To bridge these two evaluation paradigms and provide a more comprehensive assessment, ordinal versions of MAE and RMSE were additionally computed by mapping each age group to its midpoint in years, as reported in Table 7. This hybrid evaluation enables direct comparison of classification errors in temporal units (years), complementing the categorical metrics. The Custom CNN achieved both the highest classification accuracy and the lowest MAE/RMSE values, demonstrating that its predictions are not only more frequently correct but, when erroneous, deviate by smaller temporal margins than competing architectures. Nevertheless, a limitation of this approach is that it does not provide an exact chronological age estimate, and the use of class midpoints inherently constrains the resolution of the error measure to the granularity of the predefined age intervals. In this context, the findings of the present study demonstrate that deep learning models, particularly the proposed Custom CNN, achieve strong performance in dental age-group classification, highlighting their potential in addressing the limitations of traditional methods in adult populations. These results are consistent with previous studies that have reported the effectiveness of convolutional neural networks in dental image analysis and age-related classification tasks [12,13,14]. Compared to conventional approaches that rely on manual assessment, the proposed method offers improved consistency and reduced subjectivity, which are critical factors in forensic and clinical applications. The variation in performance across different age groups observed in this study may be attributed to reduced anatomical variability in older individuals, making age-related features less distinguishable. This challenge has also been noted in related studies, where the accuracy of dental age estimation decreases with increasing age due to the subtle nature of morphological changes [6,7,8]. Therefore, the application of deep learning techniques, which can capture complex and non-linear patterns in radiographic data, provides a significant advantage in such scenarios.

The dataset used in this study was specifically developed from the Egyptian population using panoramic radiographs, reflecting real clinical conditions and enhancing the practical relevance of the findings. While the dataset size is relatively limited, the proposed Custom CNN was designed to adapt effectively to the dataset characteristics, achieving stable performance compared to several pre-trained models, including YOLOv8, VGG-16, MobileNet, DenseNet-121, NASNet-Mobile, ResNet, EfficientNetV2M, EfficientNetV2, Xception, and InceptionV3.

From a clinical and forensic perspective, the proposed approach offers a practical and efficient tool for supporting age assessment, particularly in scenarios where rapid, objective, and reproducible evaluation is required. This is especially relevant in forensic investigations and legal contexts, where accuracy and consistency are essential. Moreover, the use of automated deep learning models can reduce observer bias and improve the reliability of age-group classification compared to traditional manual methods.

Despite these promising results, several challenges remain. The performance of deep learning models is highly dependent on dataset quality, size, and diversity. Variations in imaging conditions and the lack of standardized radiographic protocols may introduce inconsistencies in model training. Additionally, the risk of overfitting due to limited data and the complexity of model architectures must be carefully managed. Ethical considerations, particularly those related to patient data privacy, as well as the computational requirements of deep learning models, also represent important factors that need to be addressed. Several limitations of the present study should be acknowledged. First, the dataset was partitioned into training and validation sets only, without a fully independent held-out test set. While the validation set was kept separate from training throughout all experiments, the absence of an external test set limits the strength of generalizability claims beyond the current dataset. Cross-validation was considered as an alternative strategy; however, the computational demands of training eleven architectures under consistent conditions made this approach impractical within the present scope. Future work should address this limitation by either expanding the dataset sufficiently to allow for a three-way split or by applying k-fold cross-validation to provide more robust performance estimates. The absence of external validation on data collected from different institutions or populations is recognized as a major limitation and should be considered when interpreting the reported results. Second, although panoramic radiographs were acquired under standardized clinical conditions using a single calibrated imaging unit with controlled exposure parameters and patient positioning protocols, inter-image variability remains an inherent challenge in retrospective radiographic studies. Factors such as minor deviations in patient head positioning, differences in jaw anatomy, and natural variation in dental morphology across individuals may introduce inconsistencies that affect the feature distributions learned by the models. While data augmentation techniques, including geometric transformations, brightness adjustment, and cutout, were applied during training to enhance model robustness to such variations, their ability to fully compensate for real-world imaging variability is limited. This represents an additional constraint on the generalizability of the current findings, and future studies should consider multi-site data collection under harmonized imaging protocols to better characterize and mitigate this source of variability.

A further limitation concerns the input resolution adopted for the Custom CNN model. While all pre-trained architectures received high-resolution inputs of 640 × 640 pixels, the Custom CNN was trained on 64 × 64 pixel images to mitigate overfitting given the limited dataset size. Although this resolution proved sufficient to achieve the highest classification accuracy in the current study, it is acknowledged that spatial downsampling may result in the loss of fine-grained morphological detail—such as subtle root resorption patterns, precise pulp chamber boundaries, and early-stage alveolar bone changes—that could carry additional discriminative information for age-group classification, particularly in the older age categories where degenerative changes are subtle. Future work should investigate the effect of progressively higher input resolutions on lightweight custom CNN architectures, potentially incorporating attention mechanisms or feature pyramid networks to selectively emphasize diagnostically relevant regions without substantially increasing model complexity or overfitting risk. Future work should focus on expanding the dataset to include more diverse populations and imaging conditions, as well as exploring advanced model optimization techniques to enhance generalizability. Integrating multi-modal data and developing standardized evaluation frameworks may further contribute to improving the accuracy and reliability of dental age estimation systems.

## 6. Conclusions

Dental age-group classification in adult and senior populations represents a clinically and forensically relevant task, particularly where conventional manual methods show limited reliability. This study investigated the application of deep learning models, including a custom-designed CNN and several established pretrained architectures, for automated age-group classification from panoramic radiographic images. The proposed Custom CNN achieved the strongest overall performance among all evaluated models, demonstrating that task-specific architectures trained on domain-relevant data can effectively capture age-related dental patterns in radiographic images. Despite these encouraging results, the findings should be interpreted with caution, as they remain constrained by the dataset size, single-institution data source, absence of external validation, and the classification-based framework adopted, which does not yield exact chronological age estimates. The proposed approach is therefore best regarded at this stage as a supportive tool that complements rather than replaces traditional forensic and clinical assessment methods.

Future work should focus on external validation across diverse populations and imaging settings, as well as direct comparison with established manual forensic methods, to better establish the generalizability and practical applicability of deep learning-based dental age-group classification in real-world contexts.

## Figures and Tables

**Table 1:** The distribution of X-ray images across the age groups.

Age Group (Year)	Panoramic Image Radiograph		Individual Teeth	
	Female	Male	Female	Male
[25,30)	150	123	3690	4050
[30,40)	148	138	3996	4002
[40,50)	251	240	2571	5040
[50,60)	85	96	2210	2304
[60,70]	118	120	2360	2460
Subtotal	752	771	14,827	17,856
Total	1469		32,680	

**Table 2:** Data augmentation techniques for all models.

Augmentation Technique	Description
Rescaling	Image pixel values were scaled to the range [0, 1] by dividing by 255, ensuring standardized inputs.
Width and Height Shifts	Applied a 10% translation to both the width and height, introducing variability while retaining key features.
Zoom Range	Zoomed images within a 50% to 150% range, allowing the model to learn across different scales.
Horizontal Flip	Enabled random horizontal flipping, increasing robustness to orientation variations.
Brightness Adjustment	Modified image brightness by ±40%, helping the model adapt to different lighting conditions.
Channel Shift	Applied a minor shift of 0.015 to the color channels, adding subtle color variations.
Fill Mode	Used nearest-neighbor interpolation to fill areas created by shifts or zooms, preserving image quality.
Cutout	Random rectangular areas of the image are masked or cut out, helping the model focus on visible parts and enhancing robustness.

**Table 3:** Consistency across models: A summary of the model hyperparameters used for the parameter sweep.

Hyperparameter	Value/Setting
Number of Epochs	30
Loss Function	Categorical Cross-Entropy
Metrics	Accuracy, Precision, Recall (Sensitivity), F1-score
Rescaling	1/255
Width and Height Shifts	0.1 (10% translation)
Zoom Range	0.5 (50% to 150% scaling)
Horizontal Flip	Enabled
Brightness Range	(0.6, 1.4)
Channel Shift	0.015
Fill Mode	Nearest-neighbor interpolation
Class Weighting	“_class_weight” for class imbalance

**Table 4:** Comparing dataset results for models.

Model	Precision (%)	Recall (%)	F1-Score (%)	Accuracy (%)
**Custom CNN**	**85.0**	**80.20**	**82.50**	**85.20**
**YOLOv8**	**76.3**	**76.6**	**76.40**	**79.10**
**VGG-16**	70.87	70.37	70.05	70.00
**DenseNet-121**	67.13	67.34	56.29	67.00
**MobileNet**	64.64	64.65	64.28	65.00
**Xception**	67.68	65.32	66.20	65.00
**InceptionResNetV2**	**61.92**	**59.93**	**58.54**	**60.00**
**NASNetMobile**	61.77	58.92	58.86	59.00
**InceptionV3**	56.04	57.91	55.70	58.00
**ResNet**	51.38	56.57	49.17	57.00
**EfficientNetV2M**	43.45	49.49	41.73	49.00

**Table 5:** Performance metrics for models in different age groups.

Model	Age Group	Precision	Recall (Sensitivity)	F1-Score	Support (Samples)
Yolo8	[25,30)	0.67	0.74	0.71	55
	[30,40)	0.74	0.67	0. 71	58
	[40,50)	0.95	0.94	0.94	99
	[50,60)	0.65	0.67	0.66	37
	[60,70]	0.80	0.83	0.81	48
VGG-16	[25,30)	0.70	0.47	0.57	55
	[30,40)	0.58	0.67	0.62	58
	[40,50)	0.88	0.87	0.87	99
	[50,60)	0.47	0.49	0.48	37
	[60,70]	0.70	0.83	0.76	48
DenseNet121	[25,30)	0.76	0.47	0.58	55
	[30,40)	0.57	0.60	0.59	58
	[40,50)	0.71	0.94	0.81	99
	[50,60)	0.59	0.27	0.37	37
	[60,70]	0.67	0.75	0.71	48
MobileNet	[25,30)	0.56	0.49	0.52	55
	[30,40)	0.59	0.57	0.58	58
	[40,50)	0.77	0.87	0.82	99
	[50,60)	0.44	0.54	0.49	37
	[60,70]	0.70	0.54	0.61	48
XceptionV3	[25,30)	0.67	0.62	0.64	55
	[30,40)	0.61	0.48	0.45	58
	[40,50)	0.84	0.80	0.82	99
	[50,60)	0.36	0.57	0.44	37
	[60,70]	0.68	0.67	0.67	48
InceptionResNetV2	[25,30)	0.65	0.24	0.35	55
	[30,40)	0.46	0.72	0.56	58
	[40,50)	0.75	0.77	0.76	99
	[50,60)	0.46	0.43	0.44	37
	[60,70]	0.65	0.65	0.65	48
NASNetMobile	[25,30)	0.51	0.65	0.58	55
	[30,40)	0.69	0.34	0.46	58
	[40,50)	0.78	0.73	0.75	99
	[50,60)	0.41	0.51	0.46	37
	[60,70]	0.47	0.58	0.52	48
InceptionV3	[25,30)	0.43	0.35	0.38	55
	[30,40)	0.51	0.31	0.39	58
	[40,50)	0.66	0.91	0.76	99
	[50,60)	0.38	0.38	0.38	37
	[60,70]	0.70	0.65	0.67	48

**Table 6:** Results of the dataset for the CustomCNN model.

Class	Precision (%)	Recall (%)	F1-Score (%)	Support (%)
[25,30)	0.86	0.83	0.84	55
[30,40)	0.85	0.84	0.84	58
[40,50)	0.87	0.86	0.87	99
[50,60)	0.82	0.80	0.81	37
[60,70]	0.84	0.85	0.85	48
Macro Avg	0.85	0.84	0.84	297
Weighted Avg	0.85	0.85	0.85	297
Accuracy	0.85			297

**Table 7:** Ordinal evaluation metrics (MAE and RMSE in years) for all evaluated models, computed using age-group midpoints.

Model	Accuracy (%)	MAE (Years)	RMSE (Years)
Custom CNN	85.20	1.92	5.46
YOLOv8	79.12	2.47	6.00
VGG-16	70.37	3.91	7.84
DenseNet-121	67.34	4.57	8.72
Xception	65.32	4.46	8.30
MobileNet	64.65	4.64	8.49
InceptionResNetV2	59.93	4.97	8.63
NASNetMobile	58.92	5.88	10.19
InceptionV3	57.91	5.24	8.77
ResNet	56.57	6.55	10.73
EfficientNetV2M	49.49	8.44	12.90

## Data Availability

The data presented in this study are not publicly available due to patient privacy and ethical restrictions. Requests for access may be directed to the corresponding author.
